# The development of neurocritical care in China from the perspective of evaluation and treatment of critical neurological diseases

**DOI:** 10.3389/fneur.2023.1114204

**Published:** 2023-02-21

**Authors:** Yingying Su, Junfang Teng, Fei Tian, Jing Jing, Huijin Huang, Suyue Pan, Wen Jiang, Furong Wang, Le Zhang, Yan Zhang, Meng Zhang, Liping Liu, Jie Cao, Huaiqiang Hu, Wei Li, Cheng Liang, Liansheng Ma, Xuegang Meng, Linyu Tian, Changqing Wang, Lihua Wang, Yan Wang, Zhenhai Wang, Zhiqiang Wang, Zunchun Xie, Mingyao You, Jun Yuan, Chaosheng Zeng, Li Zeng, Lei Zhang, Xin Zhang, Yongwei Zhang, Bin Zhao, Saijun Zhou, Zhonghe Zhou

**Affiliations:** ^1^Department of Neurology, Xuanwu Hospital, Capital Medical University, Beijing, China; ^2^Department of Neurology, The First Affiliated Hospital of Zhengzhou University, Zhengzhou, China; ^3^Department of Neurology, Nanfang Hospital of Southern Medical University, Guangzhou, China; ^4^Department of Neurology, Xijing Hospital Fourth Military Medical University, Xi'an, China; ^5^Department of Neurology, Tongji Hospital, Tongji Medical College, Huazhong University of Science and Technology, Wuhan, China; ^6^Department of Neurology, Xiangya Hospital, Central South University, Changsha, China; ^7^Department of Neurology, Daping Hospital, The Army Military Medical University, Chongqing, China; ^8^Department of Neurology, Beijing Tiantan Hospital, Capital Medical University, Beijing, China; ^9^Department of Neurology, The First Hospital of Jilin University, Changchun, China; ^10^Department of Neurology, The 960(th) Hospital of Joint Logistics Support, PLA, Jinan, China; ^11^Department of Neurology, The Second Hospital of Lanzhou University, Lanzhou, China; ^12^Department of Neurology, The First Hospital of Shanxi Medical University, Taiyuan, China; ^13^Department of Neurology, The Xinjiang Uygur Autonomous Region People's Hospital, Urumqi, China; ^14^Department of Neurology, West China Hospital, Sichuan University, Chengdu, China; ^15^Department of Neurology, The First Affiliated Hospital of Anhui Medical University, Hefei, China; ^16^Department of Neurology, The Second Affiliated Hospital, Harbin Medical University, Harbin, China; ^17^Department of Neurology, Tangshan People's Hospital of Hebei Province, Tangshan, China; ^18^Neurology Center, General Hospital of Ningxia Medical University, Yinchuan, China; ^19^Department of Neurology, The First Affiliated Hospital of Fujian Medical University, Fuzhou, China; ^20^Department of Neurology, The First Affiliated Hospital of Nanchang University, Nanchang, China; ^21^Department of Neurology, The Affiliated Hospital of Guizhou Medical University, Guiyang, China; ^22^Department of Neurology, Inner Mongolia People's Hospital, Hohhot, China; ^23^Department of Neurology, The Second Affiliated Hospital of Hainan Medical University, Haikou, China; ^24^Department of Neurology, The Affiliated Hospital of Guangxi Medical University, Nanning, China; ^25^Department of Neurology, The First People's Hospital of Yunnan Province, Kunming, China; ^26^Department of Neurology, Nanjing Drum Tower Hospital, Nanjing, China; ^27^Department of Neurovascular Center, Changhai Hospital, Naval Medical University, Shanghai, China; ^28^Department of Neurology, Tianjin Medical University General Hospital, Tianjin, China; ^29^Department of Neurology, The First Affiliated Hospital of Wenzhou Medical University, Wenzhou, China; ^30^Department of Neurology, General Hospital of Northern Theater Command, Shenyang, China

**Keywords:** critical neurological disease, evaluation, treatment, NCCU, investigation

## Abstract

**Objective:**

To understand the varieties, evaluation, treatment, and prognosis of severe neurological diseases using the third NCU survey in China.

**Design:**

A cross-sectional questionnaire study. The study was completed in three main steps: filling in the questionnaire, sorting out the survey data, and analyzing the survey data.

**Results:**

Of 206 NCUs, 165 (80%) provided relatively complete information. It was estimated that 96,201 patients with severe neurological diseases were diagnosed and treated throughout the year, with an average fatality rate of 4.1%. The most prevalent severe neurological disease was cerebrovascular disease (55.2%). The most prevalent comorbidity was hypertension (56.7%). The most prevalent complication was hypoproteinemia (24.2%). The most common nosocomial infection was hospital-acquired pneumonia (10.6%). The GCS, APACHE II, EEG, and TCD were the most commonly used (62.4–95.2%). The implementation rate of the five nursing evaluation techniques reached 55.8–90.9%. Routinely raising the head of the bed by 30°, endotracheal intubation and central venous catheterization were the mostprevalent treatment strategies (97.6, 94.5, and 90.3%, respectively). Traditional tracheotomy, invasive mechanical ventilation and nasogastric tube feeding (75.8, 95.8, and 95.8%, respectively) were more common than percutaneous tracheotomy, non-invasive mechanical ventilation and nasogastric tube insertion (57.6, 57.6, and 66.7%, respectively). Body surface hypothermia brain protection technology was more commonly used than intravascular hypothermia technology (67.3 > 6.1%). The rates of minimally invasive hematoma removal and ventricular puncture were only 40.0 and 45.5%, respectively.

**Conclusion:**

In addition to traditional recognized basic life assessment and support technology, it is necessary to the use of promote specialized technology for neurological diseases, according to the characteristics of critical neurological diseases.

## Highlights

- Question: What is the current state of the Neurocritical Care Unit (NCU) in China?
- Findings: In addition to traditional recognized basic life assessment and support technology, it is necessary to promote the use of specialized technology for neurological diseases, according to the characteristics of critical neurological diseases.
- Meaning: To understand the varieties, evaluation, treatment, and prognosis of severe neurological diseases in China.

## Introduction

At the beginning of the twenty-first century, the neurocritical care committee (NCC) of the China Neurology Association (CNA) conducted national neurocritical care unit (NCCU) surveys in 2010, 2015, and 2020 (1). The results of these surveys were used to promote the rapid and orderly development of the neurocritical care specialty. The third survey was different from the first and second surveys. To align with the goal of “improving the prognosis of severe neurological diseases” in the “Neurocritical Care Society Global Partners Program,” six survey parts related to prognosis/outcome were added: types of severe neurological diseases, comorbidities, complications, nosocomial infection, evaluation technology, and treatment technology. The purpose is to guide the development of severe neurological diseases in the next 5–10 years.

## Methods

### Survey organization and form

The NCC chairman was responsible for designing the questionnaire. The NCC members were responsible for distributing and collecting the NCCU questionnaires and then verifying the first data collected in the region. The NCC survey working group (NCC-SWG) was responsible for sorting the questionnaires, checking the second data and performing the statistical analysis. The survey area covered the 31 provinces of mainland China, the autonomous regions and municipalities directly under the central government.

### Objects and methods of investigation

**Figure 1:**
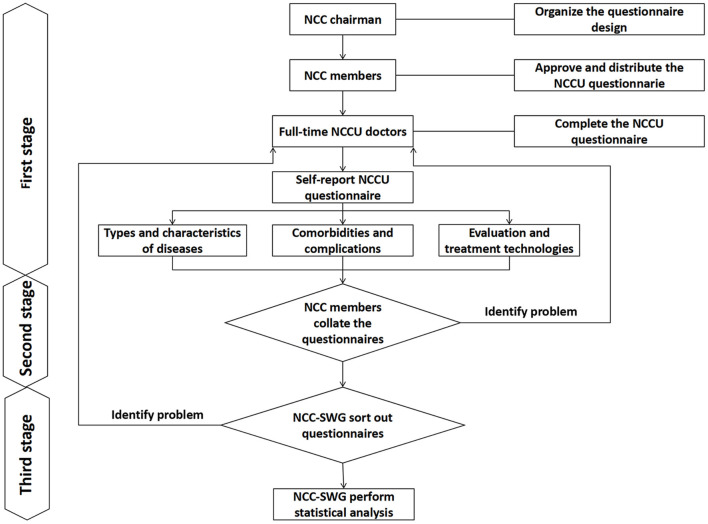
Investigation process. NCC, neurocritical care; NCCU, neurocritical care unit.

The NCCUs of tertiary hospitals employed with full-time doctors were included in the survey. The survey was a cross-sectional survey. The investigation process and steps included three stages: filling out the questionnaire, data collection and sorting, data verification and confirmation, and statistical analysis. First stage (October 1 to December 31, 2020): the NCC-SWG sent the self-report questionnaire to the regional principals (NCC members) by e-mail, and each member sent the questionnaire to the NCCU of the hospital in the region according to the survey inclusion conditions. The person in charge of the NCCU to be surveyed completed the questionnaire. Second stage (from January 1 to March 31, 2021): the person in charge of the NCCU in each region submitted a questionnaire to the NCC members. After each member received and completed the questionnaire, the questionnaire was submitted to the NCC-SWG by email. Third stage (from April 1 to June 30, 2021): the NCC-SWG first collated and verified the questionnaire (verified the query information or data by telephone or e-mail) and then performed statistical analysis on the survey results (Figure 1).

### Investigation content

The questionnaire adopted closed questions to reflect the actual situation in 2020. The survey included the types of neurological diseases, comorbidities, complications, prevalence of nosocomial infection, use of evaluation technology and treatment technology observed in the NCCU, and the questionnaire had a total of 6 parts and 57 items.

### Statistical analysis

SPSS 19.0 statistical software was used for statistical analyses. Descriptive statistics were used. The enumeration data were expressed as the number of cases (percentage), and the measurement data were expressed as the mean and range (minimum and maximum). To reflect seasonal changes, the data of the 1st month (January, April, July, and October) of each quarter in 2020 were selected, and the annual data were estimated according to the formula of monthly average sum/4^*^12.

## Results

**Figure 2:**
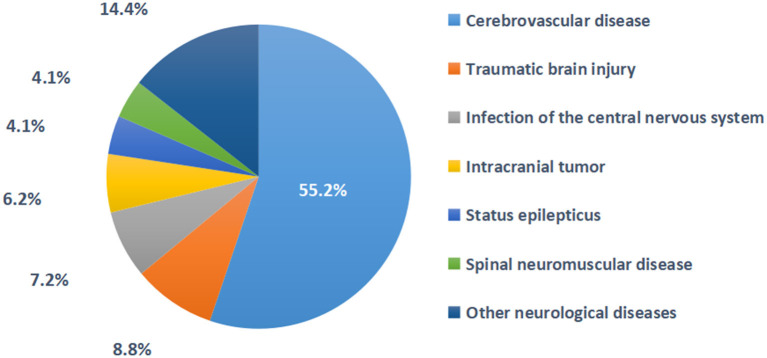
The proportion of diseases in 165 NCCU patients in 2020. Other neurological diseases include demyelinating disease, metabolic encephalopathy, postcranial/spinal surgery, etc.

**Figure 3:**
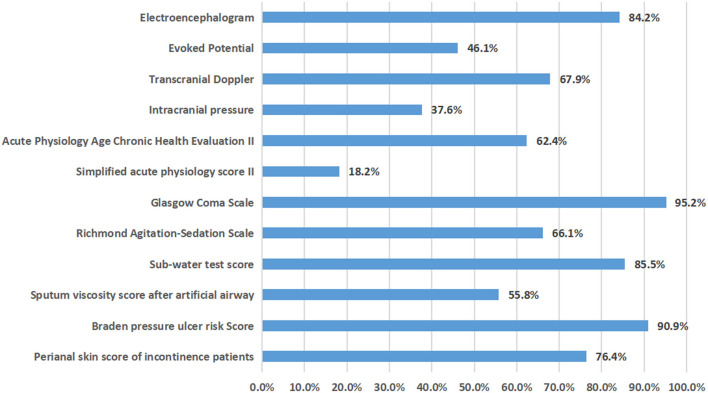
Proportion of 165 NCCU evaluation technologies in 2020.

**Figure 4:**
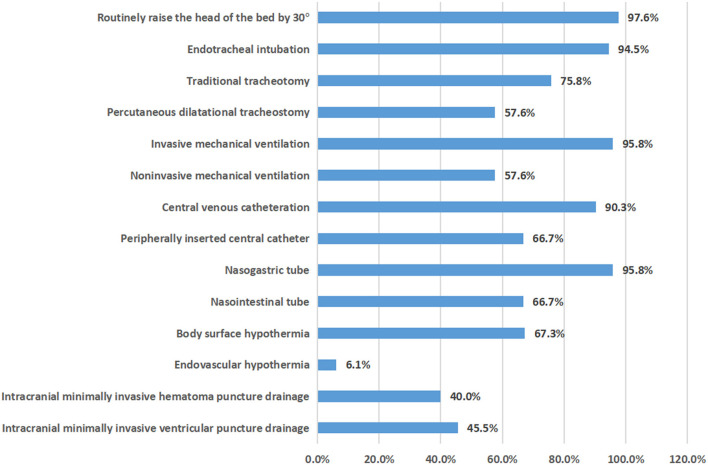
Proportion of 165 NCCU treatment technologies in 2020.

In 2020, 165 NCCUs in 152 tertiary hospitals in 29 provinces provided relatively complete information on severe neurological diseases, comorbidities, complications, nosocomial infections and mortality. It was estimated that 96,201 patients with severe neurological diseases were diagnosed and treated throughout the year (an average of 582 cases per NCCU), with an average case fatality rate of 4.1%. Among all seven types of severe neurological diseases, the three most prevalent were cerebrovascular diseases, traumatic brain injury and central nervous system infection, among which cerebrovascular diseases accounted for the highest proportion (55.2%), which was higher than the sum of all of the other severe neurological diseases (Figure 2). The different types of diseases changed in the different time periods, but they did not change significantly (Table 1). Of all six comorbidities, the three most prevalent were hypertension, diabetes and coronary heart disease (56.7, 33, and 22.7%, respectively; Supplementary material). Among the 10 complications, the four most prevalent were hypoproteinemia, hyperglycemia, acute pulmonary dysfunction and acute gastrointestinal dysfunction (24.2, 21.1, 17.5, and 14.8%, respectively). The types of comorbidities and complications changed in the different time periods, but they did not change significantly (Supplementary material). Among the four types of nosocomial infections observed, the highest proportion was hospital-acquired pneumonia (10.6%), but there was little change within the different time periods. The percent of NCCUs that had doctors and nurses in charge of nosocomial infections was as high as 87.3 and 67.3%, respectively (Supplementary material). Among the evaluation techniques, the Glasgow Coma Scale (GCS) and Acute Physiology and Chronic Health Evaluation II (APACHE II) were the most commonly used (95.2 and 62.4%). Electroencephalography (EEG) and transcranial Doppler (TCD) were the most used brain function evaluation techniques (84.2 and 67.9%). The implementation rate of the five nursing evaluation techniques reached 55.8–90.9% (Figure 3). Routinely raising the head of the bed by 30° was the most used treatment technique (97.6%). Endotracheal intubation and central venous catheterization were the second most commonly used treatment technique (94.5 and 90.3%, respectively). Traditional tracheotomy was performed more often than percutaneous tracheotomy (75.8 > 57.6%). Invasive mechanical ventilation was used more often than non-invasive mechanical ventilation (95.8 > 57.6%). Nasogastric tubes were used more often than nasointestinal tubes (95.8 > 66.7%). Body surface hypothermia was induced more often than intravascular hypothermia (67.3 > 6.1%). Minimally invasive brain hematoma removal and ventriculocentesis were performed 40.0 and 45.5%, respectively (Figure 4).

**Table 1:** Disease proportion and mortality of 165 NCCU patients in 2020^a^.

**Item**	**January**	**April**	**July**	**October**	**Annual estimation^d^**
Number of cases (average), *n*^b^	7,764 (47)	7,560 (46)	8,144 (49)	8,599 (52)	96,201 (582)
**Number of diseases (average and proportion)**, ***n*** **(*****n*****, %)**^c^					
Cerebrovascular disease	0–320 (26, 55%)	0–297 (25, 54.3%)	1–307 (27, 55.1%)	2–309 (29, 55.8%)	9–3699 (321, 55.2%)
Traumatic brain injury	0–120 (4, 8.5%)	0–140 (4, 8.7%)	0–150 (4, 8.2%)	0–140 (5, 9.6%)	0–1650 (51, 8.8%)
Infection of the central nervous system	0–27 (3, 6.4%)	0–28 (3, 6.5%)	0–32 (4, 8.2%)	0–35 (4, 7.7%)	0–366 (42, 7.2%)
Intracranial tumor	0–80 (3, 6.4%)	0–96 (3, 6.5%)	0–102 (3, 6.1%)	0–100 (3, 5.8%)	0–1134 (36, 6.2%)
Status epilepticus	0–16 (2, 4.3%)	0–25 (2, 4.4%)	0–20 (2, 4.1%)	0–18 (2, 3.8%)	0–237 (24, 4.1%)
Spinal neuromuscular disease	0–12 (2, 4.3%)	0–12 (2, 4.4%)	0–18 (2, 4.1%)	0–14 (2, 3.8%)	0–168 (24, 4.1%)
Other neurological diseases	0–90 (7, 14.8%)	0–127 (7, 15.2%)	0–119 (7, 14.2%)	0–118 (7, 13.5%)	0–633 (84, 14.4%)
Number of deaths (average, mortality), *n* (*n*, %)	0–22 (2, 4.3%)	0–18 (2, 4.3%)	0–21 (2, 4.1%)	0–20 (2, 3.8%)	0–243 (24, 4.1%)

^a^Cases from 165 NCCUs in the first month of each quarter (January, April, July, and October) in 2020 were selected to estimate the disease types and mortality of the whole year. Other neurological diseases include demyelinating disease, metabolic encephalopathy, postcranial/spinal surgery, etc.

^b^Average number of cases: number of cases/number of NCCUs.

^c^Average number of cases/average number of cases of diseases/average number of cases of death: the number of cases, cases of diseases and cases of death divided by the number of NCCUs. Proportion of diseases: the average number of diseases divided by the average total number of cases.

^d^Annual estimation: January, April, July, and October represent four quarterly sampling, respectively, take the average value and multiply by 12.

## Discussion

After the first and second surveys were administered in 2010 and 2015 in China, the third survey was administered in 2020. Unfortunately, COVID-19 was prevalent in 2020. Although NCCUs in some areas were affected by the temporary epidemic (the closure of Wuhan for 2.5 months), NCCUs in most areas still received patients with severe neurological diseases, so the size, characteristics and treatment and diagnostic ability of those NCCUs in mainland China that remained in operation was noted.

### NCCU case volume

In this study, the average volume of patients per NCCU was large (582 cases) and exceeded expectations. We speculated that this was mainly related to the high proportion of patients with cerebrovascular diseases (55.2%). The morbidity of cerebrovascular disease in China is 1,114.8 per 100,000, which is the highest in the world, and the mortality rate is 149.49 per 100,000 and is only inferior to malignant tumors and heart disease (2). As a result, the burdens on NCCU patients with severe cerebrovascular disease and the doctors and nurses who treat these patients have increased. In addition, in recent years, the use of intravascular treatment for acute stroke macrovascular occlusion has been popularized in China (3). The number of patients who received intensive monitoring in the NCCU for a short time (24–72 h) after treatment has increased, and the increase has become the main reason for the increase in the volume of NCCU patients. Therefore, the NCC suggested the expansion of NCCUs and the allocation of medical staff to allow the setup of a special area for patients with cerebrovascular diseases in the NCCU when necessary. Additionally, the NCC also suggested strengthening the monitoring and treatment of patients with severe cerebrovascular diseases to improve their short-term and long-term neurological prognosis.

### NCCU disease characteristics

Among the severe neurological diseases in this study, cerebrovascular diseases, traumatic brain injury and central nervous system infection accounted for the highest proportion (55.2, 8.8, and 7.2%), resulting in a high proportion of acute brain diseases and severe brain injury. This feature makes the focus of medical care and clinical research clearer.

In this study, hypertension, diabetes and coronary heart disease were characterized by high proportions (56.7, 33, and 22.7%, respectively). This feature was related to the high proportion of cerebrovascular diseases in the NCCU. China's national stroke statistics report showed that hypertension, diabetes and coronary heart disease were the most prevalent comorbidities that affected the prognosis (2). Therefore, in the diagnosis and treatment of primary diseases, we should pay attention to the diagnosis and treatment of comorbidities.

Among the complications observed in this study, acute pulmonary dysfunction (pulmonary infection), acute gastrointestinal dysfunction (gastric motility deficiency and acute gastric mucosal lesions), hypoproteinemia and hyperglycemia were highly prevalent (13.9, 13.4, 24.2, and 21.1%, respectively). These high prevalences were related to the high proportion of acute severe brain injury patients. Brain and multiple organ system injuries not only increase the complexity of patient monitoring and treatment but also increase the risk of death and poor prognosis regarding the patient's neurological function (4). Therefore, predicting and reducing the risk of complications have become an important part of improving prognosis.

In the NCCU, due to coma, paralysis, dysphagia, and immune impairment in patients, nosocomial infections can occur and persist. Therefore, medical staff should be vigilant in preventing and combating infection. This study showed that most of the NCCUs had doctors and nurses specifically responsible for nosocomial infections (87.3 and 67.3%), so they had a more thorough understanding of the guidelines for isolation and prevention of nosocomial infections (5) and could better implement measures to prevent and treat nosocomial infections, which led to higher satisfaction with the control of the four nosocomial infection rates, such as hospital-acquired pneumonia (2.2–10.6%).

This study did not find significant changes in the characteristics of the above diseases in the different time periods. Therefore, the NCC suggested focusing on the high proportion of patients with primary neurological diseases, comorbidities, complications and nosocomial infections, and focusing on implementing high-intensity continuous control measures.

### NCCU evaluation and treatment

In evaluating the treatment technology used for the cohort in this study, the implementation rate of EEG and TCD was very high (84.2 and 67.9%), which has further increased compared with the first survey 10 years ago (63.1 and 32.9%) (1), which was related to the change in NCCU doctors' concept of reversible or irreversible (brain death) coma evaluation and the promotion of brain death determination criteria in the past 10 years (6–8), followed by the acceptance and practice of consensus recommendations for early EEG monitoring and the treatment of status epilepticus (9). The high implementation rate of the GCS score (95.2%) was expected, and an increasing number of NCCU physicians were willing to use the APACHE II evaluation (62.4%), which allowed improvements in the cognition of patients with a brain injury and the condition of those with multiple organ systems dysfunction (10). The high implementation rate of 4/5 nursing evaluation techniques (66.1–90.9%) reflected the intensity of the transformation and promotion of the NCCU nursing management concept.

In the basic treatment technology of the NCCU in this study, traditional technologies such as endotracheal intubation, central venous catheterization and nasogastric tube feeding had a high implementation rate (>90%), which currently plays an important role, has always played an important role in the past and has provided guarantees for basic life support. The implementation rate of new technologies, such as percutaneous tracheotomy, non-invasive mechanical ventilation and naso-intestinal feeding, was <67%. Although clinical studies have confirmed that patients might benefit from these techniques (11–13), the promotion and application of these techniques still need verification in clinical practice and accurate patient selection.

In this study, the implementation rate of intravascular hypothermia was significantly lower than that of body surface hypothermia (6.1 <67.3%), and there was almost no change compared with the first survey 10 years ago. It has been reported that intravascular hypothermia treatment technology is more accurate and controllable and obtains a good neurological prognosis with fewer hypothermia complications and more reliable hypothermia efficacy (14). The implementation rate of microinvasive removal of cerebral hematoma was only 40.0%, and it has been reported that it could reduce surgical trauma and improve neurological prognosis (15). Obviously, these new technologies are not universal enough. Therefore, the NCC suggests promoting and applying advanced monitoring and evaluation technology to accurately guide treatment. Advanced life support technology and specialized neurotherapy technology should be expanded and implemented to achieve the ultimate goal of improving prognosis.

## Conclusion

Through this investigation, we have a preliminary understanding of the case volume, disease characteristics, monitoring and treatment technology and outcome/prognosis of NCCU at this stage and have put forward suggestions for reference. However, there were also some problems in this survey, such as the lack of rigorous questionnaire design and missing data, which affected the accuracy of the results. For example, (1) the estimation of mortality (outcome index) being <4.1% comes from the data submitted by each NCCU. Although we speculated that part of the reason was related to the short-term admission of patients to the NCCU due to the need for intravenous thrombolytic therapy and intravascular therapy, the questionnaire lacked relevant information. (2) The investigation items of the short-term/long-term neurological function prognosis score (prognosis index) were absent, so the main influencing factors of prognosis could not be further analyzed. Therefore, to further investigate the “goal of improving the prognosis of neurological diseases,” we also need a more careful survey scale design, more reliable NCCU information and a more reasonable data analysis to achieve the purpose of promoting development through investigation and research. In addition, the statistical data in 2020 might be affected by the temporary prevalence of COVID-19 in some parts of China. We hope that the next survey (2025) will provide more accurate data.

## Data availability statement

The original contributions presented in the study are included in the article/Supplementary material, further inquiries can be directed to the corresponding author.

## Ethics statement

The studies involving human participants were reviewed and approved by the Ethics Committee of Xuanwu Hospital, Capital Medical University, Beijing. Written informed consent from the participants or participants' legal guardian/next of kin was not required to participate in this study in accordance with the national legislation and the institutional requirements.

## Author contributions

YS: conception and design of the study and drafting the article or revising it critically for important intellectual content. JT, FT, JJ, HHua, SP, WJ, FW, LeZhang, YZ, MZ, LL, JC, HHu, WL, CL, LM, XM, LT, CW, LW, YW, ZheW, ZhiW, ZX, MY, JY, CZ, LZe, LeiZhang, XZ, YoZ, BZ, SZ, and ZZ: acquisition of data. FT, JJ, and HHua: analysis and interpretation of data. All authors: final approval of the version to be submitted.

## References

[1] Su Y-Y Wang M Feng H-H Chen W-B Ye H Gao D-Q. An overview of neurocritical care in China: A nationwide survey. Chin Med J. (2013) 126:3422–6. 24034082

[2] Wang YJ Li ZX Gu HQ Zhai Y Jiang Y Zhao XQ. China Stroke Statistics 2019 Writing Committee. China Stroke Statistics 2019: A report from the National Center for Healthcare Quality Management in Neurological Diseases. China National Clinical Research Center for Neurological Diseases, the Chinese Stroke Association, National Center for Chronic and Non-communicable Disease Control and Prevention, Chinese Center for Disease Control and Prevention and Institute for Global Neuroscience and Stroke Collaborations. Stroke Vasc Neurol. (2020) 5:211–39. 10.1136/svn-2020-000457 32826385 PMC7548521

[3] Yang P Zhang Y Zhang L Zhang Y Treurniet KM Chen W. Endovascular thrombectomy with or without intravenous alteplase in acute stroke. N Engl J Med. (2020) 382:1981–93. 10.1056/NEJMoa2001123 32374959

[4] Koennecke WB Belz W Berfelde D Endres M Fitzek S Hamilton F. Factors influencing in-hospital mortality and morbidity in patients treated on a stroke unit. Neurology. (2011) 77:965–72. 10.1212/WNL.0b013e31822dc795 21865573

[5] Siegel JD Rhinehart E Jackson M Chiarello L Health Care Infection Control Practices Advisory Committee. 2007 guideline for isolation precautions: Preventing transmission of infectious agents in health care settings. Am J Infect Control. (2007) 35(10 Suppl.2):S65–164. 10.1016/j.ajic.2007.10.007 18068815 PMC7119119

[6] Brain Brain Injury Evaluation Quality Control Center of National Health Commission Neurocritical Neurocritical Care Committe of the Chinese Society of Neurology (NCC/CSN) Neurocritical Neurocritical Care Committe of China Neurologist Association (NCC/CNA). Criteria and practical guidance for determination of brain death in adults (2nd edition). Chin Med J. (2019) 132:329–35. 10.1097/CM9.0000000000000014 30681499 PMC6595806

[7] Neurocritical Care Committee of the Chinese Society of Neurology (NCC/CSN). The Chinese expert consensus on evaluation of coma after cardiopulmonary resuscitation. Chin Med J. (2016) 129:2123–7. 10.4103/0366-6999.189054 27569242 PMC5009599

[8] Huang H Niu Z Liu G Jiang M Jia Q Li X. Early consciousness disorder in acute large hemispheric infarction: An analysis based on quantitative EEG and brain network characteristics. Neurocrit Care. (2020) 33:376–88. 10.1007/s12028-020-01051-w 32705419

[9] Tian F Su Y Chen W Gao R Zhang Y Zhang Y. RSE prediction by EEG patterns in adult GCSE patients. Epilepsy Res. (2013) 105:174–82. 10.1016/j.eplepsyres.2013.02.007 23597853

[10] Su Y-Y Li X Li S-J Luo R Ding J-P Wang L. Predicting hospital mortality using APACHE II scores in neurocritically ill patients: A prospective study. J Neurol. (2009) 256:1427–33. 10.1007/s00415-009-5129-z 19390767

[11] Sahiner IT Sahiner Y. Bedside percutaneous dilatational tracheostomy by Griggs technique: A single-center experience. Med Sci Monit. (2017) 23:4684–8. 10.12659/MSM.907006 28963447 PMC5633064

[12] Neumann B Angstwurm K Mergenthaler P Kohler S Schönenberger S Bösel J. Myasthenic crisis demanding mechanical ventilation: A multicenter analysis of 250 cases. Neurology. (2020) 94:e299–313. 10.1212/WNL.0000000000008688 31801833

[13] Wan B Fu H Yin J. Early jejunal feeding by bedside placement of a nasointestinal tube significantly improves nutritional status and reduces complications in critically ill patients vs. enteral nutrition by a nasogastric tube. Asia Pac J Clin Nutr. (2015) 24:51–7. 25740742 10.6133/apjcn.2015.24.1.03

[14] Su Y Fan L Zhang Y Zhang Y Ye H Gao D. Improved neurological outcome with mild hypothermia in surviving patients with massive cerebral hemispheric infarction. Stroke. (2016) 47:457–63. 10.1161/STROKEAHA.115.009789 26696645

[15] Lian L Xu F Hu Q Liang Q Zhu W Kang H. No exacerbation of perihematomal edema with intraclot urokinase in patients with spontaneous intracerebral hemorrhage. Acta Neurochir. (2014) 156:1735–44. 10.1007/s00701-014-2130-9 24861986

