# Supplementary figures and images for: The development of neurocritical care in China from the perspective of evaluation and treatment of critical neurological diseases
Source: Front Neurol. 2023 Feb 21;14:1114204. doi: 10.3389/fneur.2023.1114204 (PMC9990414; doi:10.3389/fneur.2023.1114204)

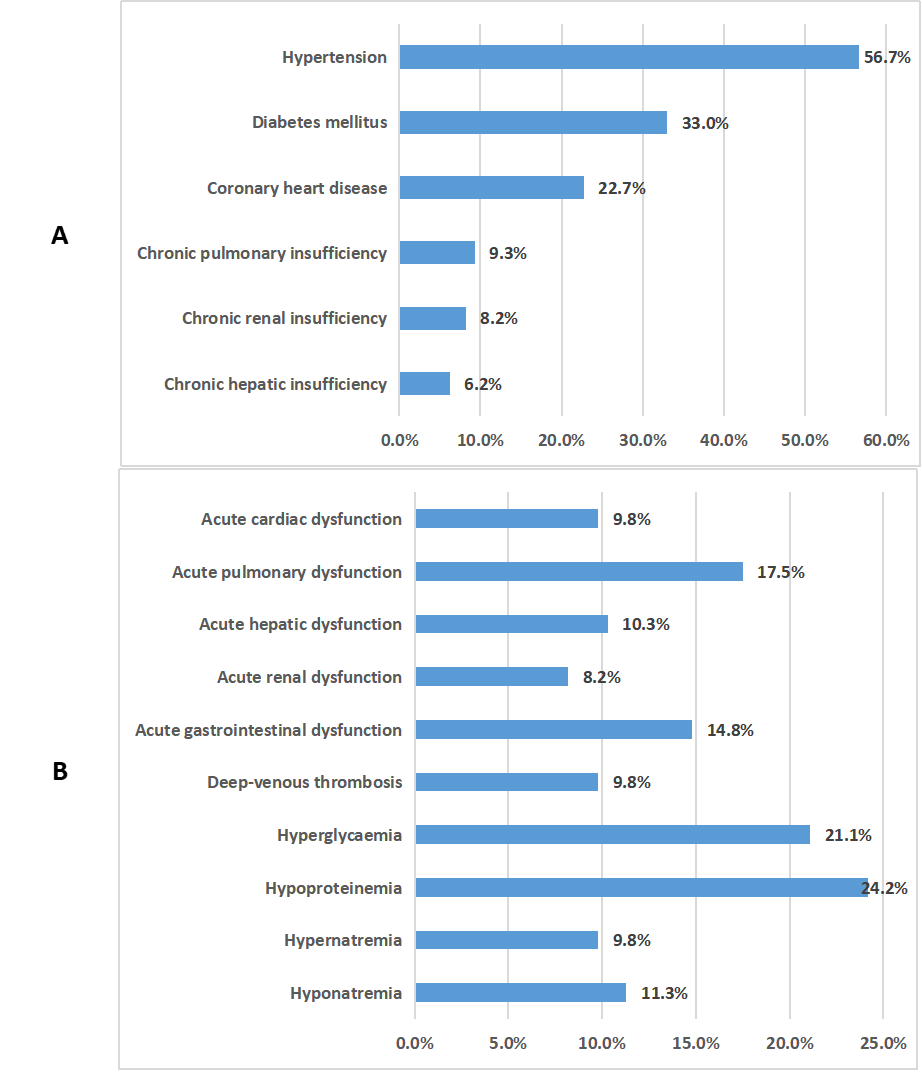

Supplement: Supplementary file 1 [file Image_1.tif]

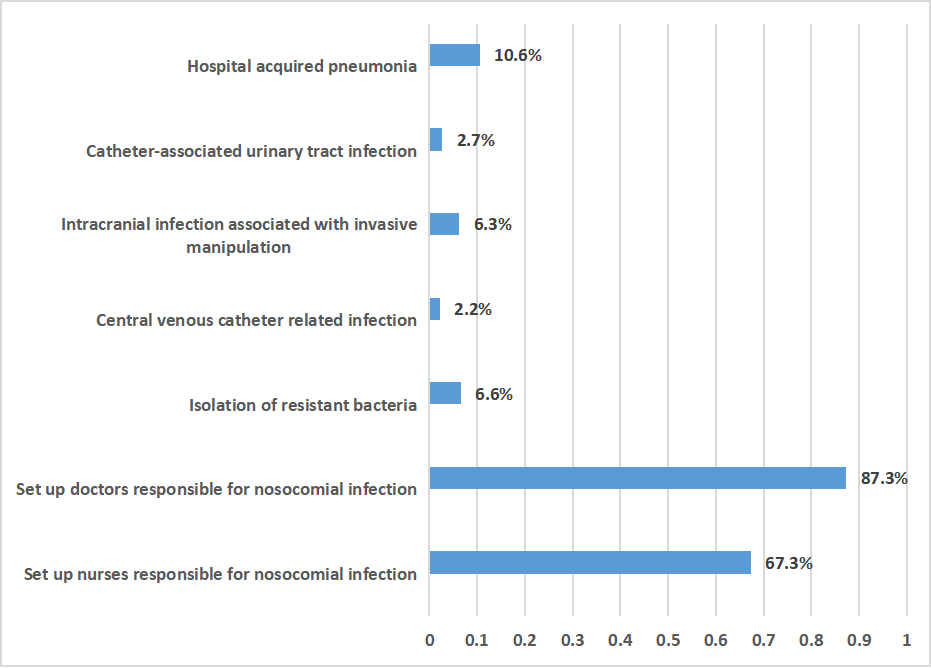

Supplement: Supplementary file 2 [file Image_2.tif]
